# The Academic Resilience Scale (ARS-30): A New Multidimensional Construct Measure

**DOI:** 10.3389/fpsyg.2016.01787

**Published:** 2016-11-18

**Authors:** Simon Cassidy

**Affiliations:** Directorate of Psychology and Public Health, University of SalfordSalford, UK

**Keywords:** resilience, academic resilience, adversity, measuring resilience, student health and wellbeing, student retention, self-efficacy, self-regulated learning

## Abstract

Resilience is a psychological construct observed in some individuals that accounts for success despite adversity. Resilience reflects the ability to bounce back, to beat the odds and is considered an asset in human characteristic terms. Academic resilience contextualizes the resilience construct and reflects an increased likelihood of educational success despite adversity. The paper provides an account of the development of a new multidimensional construct measure of academic resilience. The 30 item Academic Resilience Scale (ARS-30) explores process—as opposed to outcome—aspects of resilience, providing a measure of academic resilience based on students’ specific adaptive cognitive-affective and behavioral responses to academic adversity. Findings from the study involving a sample of undergraduate students (*N* = 532) demonstrate that the ARS-30 has good internal reliability and construct validity. It is suggested that a measure such as the ARS-30, which is based on adaptive responses, aligns more closely with the conceptualisation of resilience and provides a valid construct measure of academic resilience relevant for research and practice in university student populations.

## Introduction

### Psychological Resilience and Context-Specific Constructs

Identifying characteristics that enable academic achievement and that distinguish individuals who are successful from those who are not, setting intellectual capacity aside, remains a worthy pursuit for educational research and practice. One such characteristic is resilience. Resilience is defined by Masten et al. (1990, p. 426) as “the process of, capacity for, or outcome of successful adaptation despite challenging or threatening circumstances,” and by Riley and Masten (2005, p. 13) as “referring to patterns of positive adaptation in the face of adversity.”

On the basis that judgements about risk and adversity and evaluations of competencies and outcomes all relate directly to specific events occurring in specific contexts—in a similar way to self-efficacy beliefs (Bandura, 1997)—the existence and relevance of a unidimensional generalized global resilience construct has been questioned in favor of a multidimensional context-specific approach to resilience (Liddle, 1994; Waxman et al., 2003; Riley and Masten, 2005). As such, *academic* [sometimes *educational*] resilience has emerged as a context-specific form of individual psychological resilience and, as argued by Colp and Nordstokke (2014), was created to offer greater assessment and prediction specificity to resilience research. Closely related to individual psychological resilience, which examines the capacity for dealing with challenge and adversity, academic resilience is concerned primarily with the relevance of resilience in educational contexts and is defined as “a capacity to overcome acute and/or chronic adversity that is seen as a major threat to a student’s educational development” (Martin, 2013, p. 488). Discussing academic resilience, Martin and Marsh (2006) note that whilst there are many students who perform poorly and continue to perform poorly, there are a significant number of others who manage to turn around their academic misfortunes, flourishing and thriving despite adversity. An often-cited adversity that affects academic achievement is poverty (Kanevsky et al., 2008), and it is the capacity of some children to overcome the limitations of poverty and to succeed when others do not (Gizir, 2004), that illustrates the existence of individual resilience and underlines its importance as a psychological construct. In an academic context, resilience is characterized by those students that present with the capacity to reverse academic misfortune and failure and succeed while others continue to perform poorly and fail (Martin and Marsh, 2006).

Resilience is—universally—considered a strength or asset, a desirable and advantageous quality, characteristic or process that is likely to impact positively on aspects of an individual’s performance, achievement, health and wellbeing (Bartley et al., 2010). Martin and Marsh (2009), for instance, refer to their approach to academic resilience as an inherently asset-orientated, strength-based and aspirational approach to students’ response to academic adversity and the benefits of academic resilience are demonstrated by studies that report resilience as a significant predictor of coping at university (McLafferty et al., 2012), that report a positive relationship between academic resilience and academic achievement (Fallon, 2010), that suggest the potential to foster increased resilience through interventions (Gardynik, 2008) and that report the positive effects of educational interventions that incorporate aspects of academic resilience (Martin and Marsh, 2008). Martin and Marsh (2006) have suggested that that all students, at some point, will experience poor performance, challenge or pressure. Citing the work of Topham and Moller (2011), along with increasing suicide figures among university students (Office for National Statistics, 2013), Cheng and Catling (2015) suggest that university students have an increased vulnerability to mental illness that implies low resilience in coping with academic stress and change. Considered together, these points help to illustrate the continued relevance of academic resilience and its value as a desirable characteristic in students.

### Measuring Resilience

In accepting the value and relevance of resilience, we are faced with the task of capturing its essence in a reliable and valid construct measure. As with many latent psychological constructs, measurement of resilience has inevitably involved psychometric scales. Examples of notable resilience scales include Wagnild and Young’s (1993) Resilience Scale, the Connor-Davidson Resilience Scale (Connor and Davidson, 2003), the Resilience Scale for Adults (Friborg et al., 2003) and the Brief Resilience Scale (Smith et al., 2008). Each of these scales presents respondents with attitudinal statements constructed according to characteristics commonly associated with resilience. These include personal and social competence, acceptance of self and life, self-esteem, action-orientation, adaptability, goal-orientated strategies, problem solving, social support and family coherence, personal structure, sense of humor, endurance, and optimism and relate to the key resilience categories of dispositional attributes, family cohesion, and external supporting systems (Hoge et al., 2007). The emergent factor structure of these scales can provide further insight in to the composite elements of resilience. The Connor-Davidson Resilience Scale, for example, has been reported as yielding a five factor structure: personal competence, high standards and tenacity; trust in one’s instincts, tolerance of negative affect and strengthening effect of stress/stress-related growth; positive acceptance of change and secure personal relationships; personal control; and spiritual influences/spiritual orientation to the future, with personal competence, high standards and tenacity identified as the factor accounting for the largest proportion of variance (Connor and Davidson, 2003; see also Lamond et al., 2009).

The reported factor structure for the Connor-Davidson Resilience Scale is not fully consistent (Campbell-Sills and Stein, 2007; Lamond et al., 2009; Green et al., 2014), and with the hardiness characteristics of commitment, control and challenge also reported as features of resilience, Hoge et al. (2007) notes that despite the development of a number of scales purporting to measure resilience, there exists little consensus regarding which of these scales best captures and quantifies the construct of resilience. Lamond et al. (2009) also notes the lack of consensus on the construct definition of resilience and Hoge et al. (2007) go on to suggest that this lack of consensus is indicative of an inherent difficulty in defining the ‘notion’ of resilience; the measurement of academic resilience does not transcend this ‘inherent difficulty.’

Whilst interest in the field continues (e.g., Ricketts et al., 2015; Edwards et al., 2016), research specifically focussing on academic resilience is limited, and advances in terms of defining the construct and its associated predictive factors has been slow (Martin, 2002; Martin et al., 2010). Such advances, it is argued, are a necessary precondition for subsequent advances in construct measurement and this may, in part, explain the lack of available standardized measures of academic resilience (Cassidy, 2015). Although reference to standardized context-specific measures are rare, one popular measure—often cited in the related literature—used to measure academic resilience was presented by Martin and Marsh (2006) in their study examining educational correlates of resilience in high school children. Comprising just six items, this academic resilience subscale asks students to rate their ability to deal with setbacks, challenge, adversity and pressure in an academic setting. Individual items refer specifically to mental toughness, study stress, bouncing back from a poor mark, dealing with schoolwork pressures, confidence and dealing with such setbacks as bad marks and negative feedback. Although a brief attitudinal scale developed for use with school children, the Martin and Marsh scale arguably remains the prevalent measure of academic resilience currently available.

### Present Study

Questioning the validity of resilience measures that capture only state characteristics or positive attitudes or mood, and referring to a definition of resilience that includes reference to a response to some specific event or situation, Hoge et al. (2007) argue that resilience should in fact be measured by observing individuals during a stressful experience and assessing how well they return to normal functioning (i.e., capacity for ‘bouncing back’), “a true resilience scale measures an individual’s reaction to an experimental stress paradigm.” (Hoge et al., 2007, p. 147). Friedland (2005) also emphasizes the importance of behavioral responses (in addition to attitudinal measures) in the measurement of resilience, yet the majority of studies examining academic resilience employ generalized attitudinal response scales, mainly with samples of school children. Thus, there is an apparent lack of suitably developed standardized construct measures available to investigate academic resilience, particularly in samples of university students (Khalaf, 2014).

Waxman et al. (2003) have described resilience as referring to factors and processes that limit negative behaviors associated with stress and result in adaptive outcomes in the presence of adversity, while Morales (2008), citing McGubbin (2001), notes the discussion of resilience that considers whether it should be characterized in terms of an outcome (e.g., academic success, good grades) or process (e.g., protective factors such as a strong work ethic that helps mitigate risk and adversity). The present study offers an alternative *process*-based measure of academic resilience, focusing on adaptive and non-adaptive cognitive-affective and behavioral responses to academic adversity. Recognizing the need for significant adversity and adequate adaptability in the face of such adversity (Riley and Masten, 2005; Hoge et al., 2007), the 30 item Academic Resilience Scale (ARS-30) developed in the present study measures the responses of university students to a hypothetical, but authentic, academic adversity case vignette. The vignette was developed to portray adversity in an educational context, allowing students to respond in an adaptive or non-adaptive manner, thus providing a measure of academic resilience that is based on responses to a specific instance of academic adversity in a similar way to other resilience scales developed to capture resilience responses to specific life events (e.g., Hardy et al., 2004).

Self-regulated learning (SRL) has been conceptualized as the way in which learners control their thoughts, feelings and actions in order to achieve academically (Zimmerman and Schunk, 2001) and self-efficacy as “people’s judgments of their capabilities to organize and execute courses of action required to attain designated types of performances” (Bandura, 1997, p. 391). Both concepts are salient features of the literature related to academic resilience. Martin and Marsh (2006) for example refer closely to these concepts when proposing their 5-C model of academic resilience: confidence (self-efficacy), commitment (persistence), coordination (planning), control (how hard work and effective strategies impact achievement) and composure (low anxiety), and Newman (2002) and Sautelle et al. (2015) reported an association between high self-regulation (including adaptive help-seeking) and resilience. As such, the items comprising the ARS-30 were selected to reflect the conceptual areas of self-efficacy and self-regulation together with the range of attributes, characteristics and factors commonly associated with resilience.

A principal feature of resilience is the capacity to bounce back [from adversity], to recover and restore previous, pre-adversity, level normal functioning (Smith et al., 2008). In combining the academic adversity vignette with the presentation of associated adaptive and non-adaptive cognitive-affective and behavioral responses, it is suggested that the ARS-30 encompasses, to some degree, both the stressful experience and the quantification of the capacity to return to normal functioning posited as necessary components of a true measure of [academic] resilience (Friedland, 2005; Hoge et al., 2007). It is argued that the adversity vignette represents the critical incident from which it is necessary to recover and the degree to which adaptive responses are selected [over non-adaptive responses] confers capacity for ‘bounce back.’ The aim of the present study is to assess, evaluate and report the psychometric properties of the ARS-30 and consider its potential as a valid and reliable construct measure of academic resilience in university students.

## Materials and Methods

### Participants and Design

The sample consisted of 532 British undergraduate university students (mean age 22.4 years, *SD* = 6.2). The main analysis was conducted using a sub-group of 321 participants who completed the original vignette version of the ARS-30 (**Table 1**); the remaining participants (*n* = 211) completed the alternative vignette version of the ARS-30 that was used to assess discriminant validity [see Section The Academic Resilience Scale-30 (ARS-30)]. Participants were randomly allocated to sub-groups. The nature of the study—questionnaire development—required that a self-report questionnaire-based design with correlational and between-subjects components was employed. Academic resilience and academic self-efficacy measures were completed during a single data collection point at which time participants’ gender, age and year of study data were also recorded. Though females were overrepresented in the sample, introducing potential bias in to the data, this imbalance has been reported as representative of typical undergraduate intakes in a number of disciplines including psychology, education, subjects allied to medicine, social science, creative arts and design, veterinary science, languages and law (Bourne, 2014; Hillman and Robinson, 2016) and reflects the growing trend for females to outnumber males on two thirds of university courses (Universities and Colleges Admissions Service [UCAS], 2016) and in five sixths of higher education institutions (Hillman and Robinson, 2016).

**Table 1:** Sample details by sub-group.

Sub-group	*N*	Mean Age (*SD*)	*n*				
			Males	Females	Year 1	Year 2	Year 3
Original vignette group	321	22.4 (6.4)	56	264	237	52	31
Alternative-vignette group	211	22.5 (5.8)	34	176	157	22	32

### Materials

#### The Academic Resilience Scale-30 (ARS-30)

The aim underlying the ARS-30 was to develop a context-specific construct measure of academic resilience based on student responses to academic adversity. Scale items thus represent a sample of relevant positively and negatively phrased cognitive-affective and behavioral responses to adversity informed by, and derived from, the published literature in the fields of individual psychological resilience and academic resilience, self-regulated learning (Zimmerman and Schunk, 2001) and self-efficacy (Bandura, 1997). All items were formulated in to statements that align with accepted good practice for questionnaire design (Oppenheim, 1992; Kline, 1993). Responses to the 30 scale items were made by participants, along a 5-point Likert scale from likely (1) to unlikely (5), once they have been exposed to (i.e., had read) a short vignette. The vignette was constructed to portray an example of academic adversity, representing significant academic challenge and struggle:

You have received your mark for a recent assignment and it is a ‘fail.’ The marks for two other recent assignments were also poorer than you would want as you are aiming to get as good a degree as you can because you have clear career goals in mind and don’t want to disappoint your family. The feedback from the tutor for the assignment is quite critical, including reference to ‘lack of understanding’ and ‘poor writing and expression,’ but it also includes ways that the work could be improved. Similar comments were made by the tutors who marked your other two assignments.

Participants are asked to imagine themselves as the student characterized in the vignette and thus experiencing academic adversity. Scoring of positively phrased items was reversed so that a high ARS-30 score indicated greater academic resilience. With each of the scale items weighted equally, the global ARS-30 score, achieved by summing responses to the 30 individual items, has a theoretical range of 30–150. The scale and vignette were piloted with a group of final year undergraduate students to gather feedback on the authenticity of the vignette and the relevance of the list of 30 potential associated scale items. All 30 items were retained and no revisions were made to the vignette as a result of piloting.

For the purposes of assessing discriminate validity an alternative form of the vignette was also used in the study. The original vignette was modified so that, in its alternative form, it now depicted academic adversity being experienced by a fellow student; participants now had to complete the ARS-30 according to how they felt the student represented in the alternative vignette should respond to adversity, e.g., ‘He should keep trying’ (Cassidy, 2015):

John has received a mark for a recent assignment and it is a ‘fail.’ The marks John received for two other recent assignments were also poorer than he would want as he is aiming to get as good a degree as he can because he has clear career goals in mind and doesn’t want to disappoint his family. The feedback John received from the tutor for the failed assignment is quite critical, including reference to ‘lack of understanding’ and ‘poor writing and expression,’ but it also includes ways that the work could be improved. Similar comments were made by the tutors who marked John’s other two assignments.

#### The General Academic Self-Efficacy Scale (GASE)

The General Academic Self-Efficacy Scale (GASE) is a measure of general academic self-efficacy developed for use with university students. Participants respond to 23 statements relating to self-efficacy beliefs in an academic context according to their level of agreement with each statement, from completely disagree to completely agree using a 9-point Likert scale. Example items include: ‘I know I have the ability to complete this course successfully’; ‘I have some doubts about my ability to grasp some of the topics taught on this course’; ‘I know I have the ability to pass my examinations without too much difficulty.’ The authors of the scale report high internal (α = 0.86) and external (*r* = 0.71) reliability and suggest that significant correlations with measures of academic locus of control and computer user self-efficacy demonstrate the scale’s construct validity (Cassidy and Eachus, 2002). The GASE has a theoretical range of 23–207, with higher scores indicating greater academic self-efficacy (i.e., more positive academic self-efficacy beliefs).

### Procedure

Once informed consent had been obtained participants completed the GASE and ARS-30 together with a demographics questionnaire recording age, gender, and year of study. A sub-group of the sample (*n* = 211) completed the ASR-30 following exposure to the alternative vignette modified to depict academic adversity experienced by a fellow student instead of being personally experienced by the participant [see Section The Academic Resilience Scale-30 (ARS-30)]. The remainder of the sample (*n* = 321) completed the ARS-30 on the basis of the original—personal adversity—vignette [see Section The Academic Resilience Scale-30 (ARS-30)]. Participants were randomly assigned to the sub-groups exposed to either the alternative or original vignette. Data collection was anonymous in order to improve the validity of responses.

The study was carried out in accordance with the recommendations of both the British Psychological Society Code of Ethics and Conduct and the Research, Innovation and Academic Engagement Ethical Approval Panel, University of Salford with written informed consent from all participants in accordance with the Declaration of Helsinki.

## Results

### Descriptive Statistics

**Table 2** presents mean scores and standard deviations for each of the 30 items of the ARS-30, along with the global academic resilience score, based on the responses of a sample of undergraduate students (*n* = 321). For all items, a higher score (range 1–5) indicates greater agreement with the statement. The global ARS-30 score represents the summation of responses to the 30 individual items, with a higher global score (theoretical range 30–150) reflecting greater academic resilience.

**Table 2:** Mean score and standard deviation for individual academic resilience items Academic Resilience Scale (ARS-30).

Item	Mean ±*SD*
(1) I would not accept the tutors’ feedback	4.26 ± 1.11
(2) I would use the feedback to improve my work	4.75 ± 0.55
(3) I would just give up	4.47 ± 0.86
(4) I would use the situation to motivate myself	4.13 ± 1.02
(5) I would change my career plans	4.14 ± 0.98
(6) I would probably get annoyed	2.54 ± 1.21
(7) I would begin to think my chances of success at university were poor	3.17 ± 1.12
(8) I would see the situation as a challenge	3.88 ± 1.03
(9) I would do my best to stop thinking negative thoughts	3.84 ± 1.02
(10) I would see the situation as temporary	3.70 ± 1.02
(11) I would work harder	4.61 ± 0.78
(12) I would probably get depressed	3.22 ± 1.22
(13) I would try to think of new solutions	4.17 ± 0.82
(14) I would be very disappointed	1.83 ± 1.12
(15) I would blame the tutor	4.31 ± 0.93
(16) I would keep trying	4.52 ± 0.72
(17) I would not change my long-term goals and ambitions	4.13 ± 0.98
(18) I would use my past successes to help motivate myself	4.26 ± 0.94
(19) I would begin to think my chances of getting the job I want were poor	3.38 ± 1.17
(20) I would start to monitor and evaluate my achievements and effort	3.99 ± 0.96
(21) I would seek help from my tutors	4.30 ± 0.99
(22) I would give myself encouragement	4.03 ± 0.96
(23) I would stop myself from panicking	3.41 ± 1.15
(24) I would try different ways to study	4.03 ± 0.95
(25) I would set my own goals for achievement	4.13 ± 0.85
(26) I would seek encouragement from my family and friends	3.77 ± 1.31
(27) I would try to think more about my strengths and weaknesses to help me work better	4.05 ± 0.93
(28) I would feel like everything was ruined and was going wrong	3.47 ± 1.23
(29) I would start to self-impose rewards and punishments depending on my performance	2.84 ± 1.22
(30) I would look forward to showing that I can improve my grades	4.27 ± 0.91
Global ARS-30 score	115.61 ± 14.78

### Factor Structure

**FIGURE 1:**
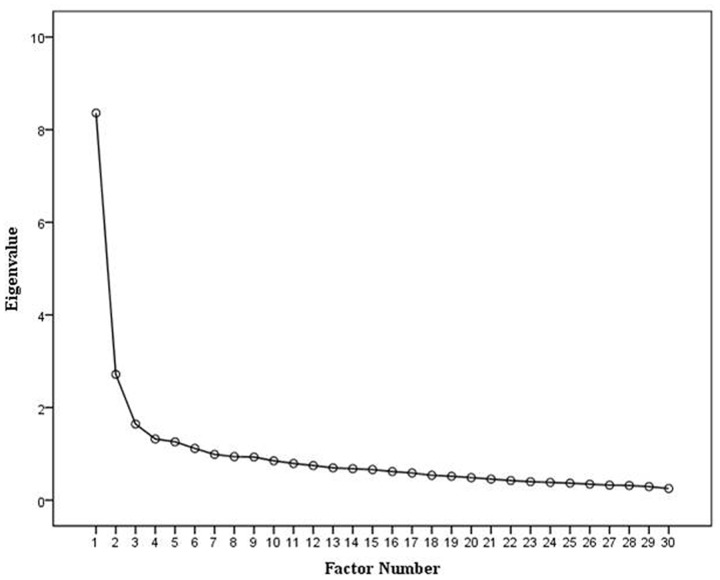
**Scree plot for the ARS-30 showing the amount of variance accounted for by each factor**.

The scree plot presented in **Figure 1** indicates that there are three meaningful factors for extraction, including factor 3 located at the point of inflection (Cattell, 1966; Field, 2013). Initial retention of three factors is supported by the total variance of 42.4% accounted for by the three factors: 27, 9.1, and 5.5%, respectively. Given that the sample size exceeds 200 the scree plot is considered a reliable basis for factor selection (Stevens, 2002; Field, 2013). Equally, retention of the three factors meets Kaiser’s (1960) criterion for retaining factors with eigenvalues greater than 1(factor 1 = 8.359, factor 2 = 2.716, factor 3 = 1.644), although Kaiser’s criteria was not the primary criterion for selection given the suggestion that this can overestimate the number of factors to be retained (Field, 2013). Sampling adequacy was verified by KMO = 0.897 (Kaiser, 1970; Hutcheson and Sofroniou, 1999, as cited in Field, 2013). Bartlett’s test of sphericity (χ^2^ = 3457.39, *df* = 435, *p* < 0.001) and determinant of *R*-matrix > 0.00001 indicate that inter-variable correlations are suitable for factor analysis (Field, 2013).

**Table 3** shows factor loadings after maximum likelihood oblique (promax) rotation, with loading of 0.3 and above in bold (Field, 2013). Item clustering suggests that factor 1 (items 1, 2, 3, 4, 5, 8, 9, 10, 11, 13, 15, 16, 17, and 30) represents perseverance, factor 2 (items 18, 20, 21, 22, 24, 25, 26, 27, and 29) reflecting and adaptive help-seeking and factor 3 (items 6, 7, 12, 14, 19, 23, and 28) negative affect and emotional response.

**Table 3:** Maximum likelihood estimates of the oblique (promax) rotated factor loadings for the ARS-30.

ARS-30 Item		Factor loadings		
		Factor 1	Factor 2	Factor 3
**Factor 1 (Perseverance)**:				
(11) I would work harder		**0.776**	-0.013	-0.174
(16) I would keep trying		**0.758**	-0.008	-0.046
(2) I would use the feedback to improve my work		**0.685**	-0.069	-0.129
(3) I would just give up		**0.629**	-0.136	0.242
(13) I would try to think of new solutions		**0.629**	0.096	-0.085
(5) I would change my career plans		**0.585**	-0.247	0.080
(4) I would use the situation to motivate myself		**0.505**	0.030	0.079
(17) I would not change my long-term goals and ambitions		**0.502**	-0.053	-0.033
(8) I would see the situation as a challenge		**0.427**	0.154	-0.097
(30) I would look forward to showing that I can improve my grades		**0.429**	0.242	0.047
(10) I would see the situation as temporary		**0.384**	0.033	0.182
(9) I would do my best to stop thinking negative thoughts		0.290	0.161	0.254
(15) I would blame the tutor		0.260	0.158	0.168
(1) I would not accept the tutors’ feedback		0.146	-0.063	0.103
**Factor 2 (Reflecting and adaptive help-seeking):**				
(27) I would try to think more about my strengths and weaknesses to help me work better		-0.046	**0.823**	-0.098
(22) I would give myself encouragement		-0.010	**0.707**	0.145
(26) I would seek encouragement from my family and friends		-0.211	**0.580**	-0.024
(24) I would try different ways to study		0.104	**0.571**	0.033
(25) I would set my own goals for achievement		0.212	**0.563**	-0.026
(21) I would seek help from my tutors		0.055	**0.448**	0.016
(20) I would start to monitor and evaluate my achievements and effort		0.322	**0.356**	-0.144
(29) I would start to self-impose rewards and punishments depending on my performance		-0.101	**0.323**	-0.207
(18) I would use my past successes to help motivate myself		0.317	**0.321**	0.095
**Factor 3 (Negative affect and emotional response):**				
(28) I would feel like everything was ruined and was going wrong		0.049	0.015	**0.730**
(7) I would begin to think my chances of success at university were poor		0.093	-0.074	**0.672**
(12) I would probably get depressed		0.058	-0.107	**0.669**
(14) I would be very disappointed		-0.386	-0.001	**0.657**
(19) I would begin to think my chances of getting the job I want were poor		0.144	-0.085	**0.635**
(6) I would probably get annoyed		-0.138	0.074	**0.505**
(23) I would stop myself from panicking		0.073	0.281	**0.386**

**KMO**	**Bartlett’s sphericity**	**Determinant**		
**0.9**	**χ^2^ = 3457.39*, p* < 0.001**	**1.36**		

Eigenvalues		8.36	2.72	1.64
% of variance		27.86	9.05	5.48
Cronbach’s α		0.83	0.78	0.80

The inter factor correlations presented in **Table 4** indicate medium to large positive correlations between the three factors. Increased perseverance was associated with increased reflecting and adaptive help-seeking (*r* = 0.71) and increased avoidance of negative affective and emotional response (*r* = 0.45); increased reflecting and adaptive help-seeking was associated with increased avoidance of negative affective and emotional response (*r* = 0.39).

**Table 4:** Eigenvalues, percentage of explained variance, inter-factor correlations and factor-total correlations for the ARS-30.

	Eigenvalue	Percentage explained variance	Inter-factor correlations		
			Factor 1	Factor 2	Factor 3
Factor 1	8.36	27.86	–	–	–
Factor 2	2.72	9.05	0.71	–	–
Factor 3	1.64	5.48	0.45	0.39	–

**Table 5** presents mean scores and standard deviations for each of the three factors of the ARS-30, perseverance, reflecting and adaptive help-seeking, and negative affect and emotional response based on the responses from a sample of undergraduate students (*n* = 321). For each factor, the factor score represents the summation of responses to the individual items loading highest on that factor (**Table 3**), with higher scores reflecting more adaptive responses for each factor.

**Table 5:** Mean and standard deviation ARS-30 scores by factor.

Factor	No. of Items	Theoretical range	Mean ± SD
Perseverance	14	14–70	59.17 ± 7.22
Reflecting and adaptive help-seeking	9	9–45	35.41 ± 5.57
Negative affect and emotional response	7	5–35	21.04 ± 5.53

### Reliability Analysis

Item-scale analysis is presented in **Table 6** for the ARS-30. Cronbach’s α of 0.90 indicated high internal consistency reliability for the global scale (i.e., summation of the 30 items). All item-total correlations were above 0.3 (Field, 2013) with the exception of items 1 (0.14) and 14 (0.12); as deletion of these items does not increase the overall Cronbach’s α it is suggested that all items are contributing positively to the scale’s reliability, supporting the case for retaining these items (Field, 2013). Cronbach’s α was also acceptable for each of the three retained factors: factor 1 α = 0.83; factor 2 α = 0.78; and factor 3 α = 0.80. Item-total correlations ranged between 0.41 and 0.63 for factor 1(with the exception of item 1 = 0.11), between 0.37 and 0.65 for factor 2 (with the exception of item 29 = 0.15), and between 0.45 and 0.65 for factor 3. ‘Alpha if item deleted’ results indicate that deleting items 1 and 29 would increase—marginally—the reliability of factors 1 (by 0.017) and 2 (by 0.031) respectively, potentially raising questions regarding the retention of these items, at least in instances where factor-based subscales are to be utilized in the application of the ARS-30.

**Table 6:** Item-scale analysis of the ARS-30.

Item		Corrected item-scale correlation	Cronbach’s α if item is deleted
(1) I would not accept the tutors’ feedback		0.14	0.89
(2) I would use the feedback to improve my work		0.44	0.89
(3) I would just give up		0.58	0.88
(4) I would use the situation to motivate myself		0.51	0.88
(5) I would change my career plans		0.35	0.89
(6) I would probably get annoyed		0.30	0.89
(7) I would begin to think my chances of success at university were poor		0.48	0.88
(8) I would see the situation as a challenge		0.43	0.89
(9) I would do my best to stop thinking negative thoughts		0.56	0.88
(10) I would see the situation as temporary		0.48	0.88
(11) I would work harder		0.52	0.88
(12) I would probably get depressed		0.41	0.89
(13) I would try to think of new solutions		0.54	0.88
(14) I would be very disappointed		0.12	0.89
(15) I would blame the tutor		0.48	0.88
(16) I would keep trying		0.59	0.88
(17) I would not change my long-term goals and ambitions		0.36	0.89
(18) I would use my past successes to help motivate myself		0.58	0.88
(19) I would begin to think my chances of getting the job I want were poor		0.49	0.88
(20) I would start to monitor and evaluate my achievements and effort		0.47	0.88
(21) I would seek help from my tutors		0.40	0.89
(22) I would give myself encouragement		0.64	0.88
(23) I would stop myself from panicking		0.53	0.88
(24) I would try different ways to study		0.57	0.88
(25) I would set my own goals for achievement		0.61	0.88
(26) I would seek encouragement from my family and friends		0.25	0.89
(27) I would try to think more about my strengths and weaknesses to help me work better		0.54	0.88
(28) I would feel like everything was ruined and was going wrong		0.54	0.88
(29) I would start to self-impose rewards and punishments depending on my performance		0.03	0.90
(30) I would look forward to showing that I can improve my grades		0.58	0.88
**Internal consistency of the ARS-30**			
Factor 1	α = 0.83		
Factor 2	α = 0.78		
Factor 3	α = 0.80		
Global Scale	α = 0.90		

### Validity Analysis

Higher global academic resilience scores were associated with increased academic self-efficacy (*r* = 0.49, *N* = 319, *p* < 0.01) and increased age (*r* = 0.20, *N* = 317, *p* < 0.01; **Table 7**). That separate independent analyses of the factors did not result in higher correlations with academic self-efficacy than analysis of the global score may indicate greater utility of the ARS-30 as a unidimensional measure, with less emphasis on the scale’s multidimensional properties unless these there is clear focus on these in the scale’s application (Sánchez-López and Dresch, 2008). Additionally, robust correlations between factors (reported in **Table 4**) indicate the existence of a shared psychological variable common across factors, so that factor scores can be meaningfully combined to form a global academic resilience score (Furr, 2011).

**Table 7:** Correlation coefficients between ARS-30 and General Academic Self-Efficacy Scale (GASE).

ARS-30	GASE		
	*r*	*p*	*N*
Global score	0.49	<0.01	319
Factor 1	0.48	<0.01	320
Factor 2	0.35	<0.01	320
Factor 3	0.31	<0.01	319

Although there were mean differences in academic resilience scores between male and female students and between first, second and third year students (**Table 8**), these differences did not reach statistical significance (*p* > 0.05). Whilst unequal sample sizes, as seen here, may increase the potential risk of errors, the risk is reduced provided, as is the case here, the homogeneity of variance assumption is met. Nevertheless, inferential findings related to gender and year of study inter-group comparisons should be interpreted with caution.

**Table 8:** Mean ARS-30 scores by gender and year of study.

Mean ARS-30 score ±*SD*				
Male (*n* = 56)	Female (*n* = 262)	Year 1 (*n* = 235)	Year 2 (*n* = 52)	Year 3 (*n* = 31)
113.46 ± 15.73	116.03 ± 14.58	116.22 ± 15.12	115.13 ± 13.41	111.45 ± 14.36

When the ARS-30 was completed in response to an alternative form of the original adversity vignette, varied to describe adversity experienced by a fellow student [see Section The Academic Resilience Scale-30 (ARS-30)], significant differences in mean global ARS-30 scores emerged with large effect size (Cohen, 1988) (*t* = 11.27, *df* = 525, *p* < 0.001, *d* = 0.98), providing evidence supporting the scale’s discriminant validity (**Table 9**). A comparison of mean GASE scores across the two vignette groups (Original Group *M* = 145.78 [*SD* = 19.3], Alternative Group *M* = 146.37 [*SD* = 19.4] did not reveal a significant difference (*t* = 0.341, *df* = 529, *p* > 0.05)), indicating that differences in ARS-30 scores resulted from manipulation of the vignette and not group differences in academic self-efficacy.

**Table 9:** Mean global ARS-30 score by vignette group.

Mean ARS-30 Score ±*SD*	
Original vignette group (*n* = 319)	Alternative vignette group (*n* = 208)
115.61 ± 14.78	128.54 ± 11.46

## Discussion

In accepting the argument that resilience is not a unitary construct, existing instead as a context-specific multidimensional construct (Liddle, 1994; Waxman et al., 2003; Riley and Masten, 2005), and in light of continued interest in studying resilience in student populations (e.g., Ricketts et al., 2015; Edwards et al., 2016) yet limited options for construct measurement (Cassidy, 2015), the present study sought to develop a context-specific construct measure of academic resilience and report salient psychometric properties related to evaluation of the measure. In particular, there was an attempt to respond to the suggestion that resilience relates to a specific event (Hoge et al., 2007) and that any measure of resilience should include behavioral responses to that event (Friedland, 2005). The thirty-item Academic Resilience Scale (ARS-30) is a context-specific measure of academic resilience comprising cognitive-affective and behavioral responses to adversity in an academic setting. Scale items are drawn from theoretically relevant concept domains including self-efficacy and self-regulated learning and reflect commonly cited definitions and dispositional attributes associated with psychological resilience (Hoge et al., 2007).

### Factor Structure

Exploratory factor analysis was conducted to investigate the factor structure of the ARS-30. Three factors emerged: factor 1, interpreted as *perseverance;* factor 2, interpreted as *reflecting and adaptive-help-seeking;* and factor 3, interpreted as *negative affect and emotional response*. The emerging factors accounted for a total of 42.4% of variance in academic resilience scores and resemble factors previously reported in studies focussing on the measurement of resilience (e.g., Connor and Davidson, 2003; Hoge et al., 2007; Lamond et al., 2009) as well as reflecting aspects of self-regulation and self-efficacy. The most important factor was perseverance, accounting for 27% of variance. This was followed by reflecting and adaptive-help-seeking, accounting for 9.1% of variance, and finally negative affect and emotional response accounting for 5.5% of variance. Some authors have suggested that if the largest emerging factor accounts for three times the variance of that of the subsequent factor, the construct measure can be considered unidimensional (Gorsuch, 1983). Equally, robust correlations between the dimensions, as reported here for the ARS-30, mean that dimension scores can be combined to represent a meaningful unitary global academic resilience score (Furr, 2011). However, as the emerging factors reflect previously identified and meaningful aspects of resilience (e.g., Wagnild and Young, 1993; Connor and Davidson, 2003; Martin and Marsh, 2006), and because the primary purpose of the ARS-30 is to facilitate interventions aimed at building academic resilience (Cassidy, 2015), it is suggested that there remains significant value, dependent up on the intended application, in utilizing the multidimensionality of the scale. A similar approach to dimensionality has been suggested for other psychometric instruments such as the Multidimensionality Self-Esteem Inventory (O’Brien and Epstein, 1988) which Furr (2011) report as assessing several correlated dimensions of self-esteem which can be both scored separately or combined to form a total social self-esteem score.

Factor 1, perseverance, includes items featuring hard work and trying, not giving up, sticking to plans and goals, accepting and utilizing feedback, imaginative problem solving and treating adversity as an opportunity to meet challenges and improve as central themes. There are clear parallels between this factor and factors previously identified, including perseverance (involving persistence despite adversity, willingness to continue to struggle and to practice self-discipline, Wagnild and Young, 1993), personal control and tenacity (Connor and Davidson, 2003), commitment and control (i.e., persistence, hard work and effective strategies, Martin and Marsh, 2006), and personal control and goal orientation (Lamond et al., 2009). Items loading on factor 2, reflecting and adaptive-help-seeking, features themes including reflecting on strengths and weakness, altering approaches to study, seeking help, support and encouragement, monitoring effort and achievements and administering rewards and punishments. As with factor 1, there are evident parallels between factor 2 and previously reported factors including self-reliance (belief in one’s capabilities and recognizing personal strengths and limitations) reported by Wagnild and Young (1993), adaptability reported by Lamond et al. (2009) and adaptive help-seeking reported by Newman (2002). Finally, factor 3, negative affect and emotional response features themes including anxiety, catastrophising, avoiding negative emotional responses, optimism and hopelessness and is similar to acceptance of negative affect reported by Connor and Davidson (2003) and Lamond et al. (2009), composure (low anxiety) reported by Martin and Marsh (2006) and meaningfulness (the belief that one has purpose in life and something to live for) reported by Wagnild and Young (1993).

Each of the emerging factors represents common features evident in existing research studies investigating resilience, with clear similarities and overlaps with concepts and constructs identified as relevant in previous studies of general and context-specific resilience. Thus, the emerging factor structure, and the degree to which it relates to accepted theoretical definitions and relevant constituents of resilience, supports the construct validity of the ASR-30 and the notion of academic resilience as a context-specific—multidimensional—resilience construct.

### Reliability

Item analysis presented convincing evidence for the internal consistency reliability of the scale, with the reported Cronbach’s alpha of 0.90 exceeding levels normally considered acceptable (Cronbach, 1990; Field, 2013). Equally acceptable alphas between 0.78 and 0.83 were reported for factor level reliability analysis. Low item-total correlations did raise doubts regarding the functioning of three items (items, 1, 14, and 29). However, as deletion of these items did not raise the overall reliability of the global scale, and did so only marginally at factor level, they were retained on the basis that all items contributed positively to the internal reliability of the scale (Field, 2013).

### Validity

Previous studies have reported significant associations between resilience and theoretically relevant constructs including self-efficacy (Hamill, 2003; Martin and Marsh, 2006, 2008). The significant positive correlation between ARS-30 scores and academic self-efficacy (*r* = 0.49) reported in the present study serves to demonstrate the concurrent validity of the scale. The discriminant validity of the scale was supported by significant mean differences and large effect size (*d* = 0.98) in ASR-30 responses to two independent versions of the academic adversity vignette (*p* < 0.001), which was not explained by group differences in academic self-efficacy (*p* > 0.05). Findings from previous studies examining the relationship between resilience and age, gender and experience have been mixed (e.g., Martin and Marsh, 2006, 2008; Allan et al., 2014; Khalaf, 2014). As such, the weak but significant negative correlation between ARS-30 scores and age (*r* = 0.20), along with small but non-significant (*p* > 0.05) gender and experience differences in mean ARS-30 scores reported in the present study offer no clearly interpretable additional evidence regarding the validity of the ARS-30. That scale items were selected to reflect generally accepted definitions, theoretical understandings and factors, constructs and attributes commonly associated with resilience is presented as evidence of the Scale’s content validity (Wagnild and Young, 1993).

## Conclusion

Whilst the psychometric properties reported here are convincing and support the ARS-30 as a construct measure of academic resilience, further developmental work in several areas is needed, particularly involving assessment of academic resilience across a number of data points in order to establish the test-retest reliability and predictive validity of the scale.

The degree to which the ARS-30 captures ‘bounce back’ or recovery from the challenge of academic adversity also needs further evaluation. While it is suggested that ARS-30 scores reflect the capacity for bounce back, this can only be fully established once findings from studies involving recovery to original functioning—*actual* bounce back—are available. Quantifying and calculating actual bounce back can however be problematic, requiring pragmatism, as the basis for assessment and measurement is likely to shift according to the particular study or practice parameters, as well as—ideally—the need for the existence of a pre-adversity baseline functioning measure against which to compare post-adversity functioning to determine recovery. Additionally, temporal trajectories of resilience can be complex, so bounce back may not be immediate but instead occur over a period of time post adversity (e.g., Bonanno et al., 2015). Studies exploring associations between ARS-30 scores and existing measures of resilience that specifically target bounce back, such as the Brief Resilience Scale (Smith et al., 2008), may help gain further insight in to the capacity of the ARS-30 to capture bounce back as a feature of academic resilience.

Equally, given the underrepresentation of males in the sample used in the present study, there is need to conduct further studies that specifically address the issue of generalizability of the ARS-30 to male students. Advances in psychometric theory which are currently emerging in the field, such as Generalizability Theory, may also offer greater insight in to potential sources of measurement error that is particularly pertinent to applied assessment contexts, such as universities and schools, as in the case of the ARS-30 (Briesch et al., 2014).

Nevertheless, the ARS-30 represents a unique and novel approach to the measurement of academic resilience in university students. It is argued that the measure offers validity beyond that offered by existing generalized attitudinal measures of resilience that capture only state attributes and mood (Hoge et al., 2007). Because the ARS-30 measures cognitive-affective and behavioral responses to instances of academic adversity, representing positive enabling factors such as sense of mastery, belief that one’s efforts can make a difference and effective approaches to learning (Bandura, 2006)—that Newman and Blackburn (2002) state have been under researched in the context of resilience—, together with assessment of emotional responses to adversity, believed to facilitate or impede resilience (Fredrickson, 2001), it is suggested that it can be utilized in both research and practice as a diagnostic measure, identifying non-adaptive responses to academic adversity and helping inform interventions aimed at developing resilience in students. Martin and Marsh (2009) have already proposed that students can learn to be more academically resilient through the development of positive cognitive, affective and behavioral orientations to school and academic life, which Martin and Marsh go on to suggest may be more effectively achieved by increasing individuals’ exposure to protective and enabling factors. Edwards et al. (2016) point out that as it is not possible to control the extent to which individual students are exposed to adversity, the focus should be on interventions aimed at improving resilience in those at risk of negative outcomes associated with adverse experiences. The ARS-30, it is suggested, has the potential to help identify limitations in existing student responses to academic adversity and to assist the development of interventions aimed at fostering adaptive responses, and to provide a measure of the efficacy of such interventions in terms of developing students’ academic resilience.

## Author Contributions

The contribution of author SC meets the following criteria: Substantial contributions to the conception or design of the work; or the acquisition, analysis, or interpretation of data for the work; and Drafting the work or revising it critically for important intellectual content; and Final approval of the version to be published; and Agreement to be accountable for all aspects of the work in ensuring that questions related to the accuracy or integrity of any part of the work are appropriately investigated and resolved.

## Conflict of Interest Statement

The author declares that the research was conducted in the absence of any commercial or financial relationships that could be construed as a potential conflict of interest.
